# Acute effect of fine particulate matter on blood pressure, heart rate and related inflammation biomarkers: A panel study in healthy adults

**DOI:** 10.1016/j.ecoenv.2021.113024

**Published:** 2021-12-25

**Authors:** Zhaoyuan Li, Yisi Liu, Tianjun Lu, Shouxin Peng, Feifei Liu, Jinhui Sun, Hao Xiang

**Affiliations:** a Department of Global Health, School of Public Health, Wuhan University, 115# Donghu Road, Wuhan 430071, China; b Global Health Institute, School of Public Health, Wuhan University, 115# Donghu Road, Wuhan 430071, China; c Department of Environmental and Occupational Health Sciences, University of Washington, Seattle, WA 98105, USA; d Department of Earth Science and Geography, California State University Dominguez Hills, 1000 E. Victoria St, Carson, CA 90747, USA

**Keywords:** Individual PM_2.5_ exposure, Acute effect, Heart rate, Fibrinogen, Mediation effect

## Abstract

Epidemiological evidence of short-term fine particulate matter (PM_2.5_) exposure on blood pressure (BP), heart rate (HR) and related inflammation biomarkers has been inconsistent. We aimed to explore the acute effect of PM_2.5_ on BP, HR and the mediation effect of related inflammation biomarkers. A total of 32 healthy college students were recruited to perform 4 h of exposure at two sites with different PM_2.5_ concentrations in Wuhan between May 2019 and June 2019. The individual levels of PM_2.5_ concentration, BP and HR were measured hourly for each participant. Blood was drawn from each participant after each visit and we measured the levels of inflammation markers, including serum high-sensitivity C-reactive protein and plasma fibrinogen. Linear mixed-effect models were to explore the acute effect of PM_2.5_ exposure on BP, HR, and related inflammation biomarkers. In addition, we evaluated related inflammation biomarkers as the mediator in the association of PM_2.5_ and cardiovascular health indicators. The results showed that a 10 μg/m^3^ increment in PM_2.5_ concentration was associated with an increase of 0.84 (95% CI: 0.54, 1.15) beats/min (bpm) in HR and a 3.52% (95% CI: 1.60%, 5.48%) increase in fibrinogen. The lag effect model showed that the strongest effect on HR was observed at lag 3 h of PM_2.5_ exposure [1.96 bpm (95% CI: 1.19, 2.75)], but for fibrinogen, delayed exposure attenuated the association. Increased fibrinogen levels may account for 39.07% (P = 0.44) of the elevated HR by PM_2.5_. Null association was observed when it comes to short-term PM_2.5_ exposure and BP. Short-term exposure to PM_2.5_ was associated with elevated HR and increased fibrinogen levels. But our finding was not enough to suggest that exposure to PM_2.5_ might induce adverse cardiovascular effects by the pathway of inflammation.

## Highlights

• PM_2.5_ exposure is a risk factor for cardiovascular diseases.
• Personal exposure to PM_2.5_ for a short term can elevate heart rate and fibrinogen levels.
• The role of inflammation in PM_2.5_-induced cardiovascular effects was explored.

## Graphical Abstract

**Figure:**
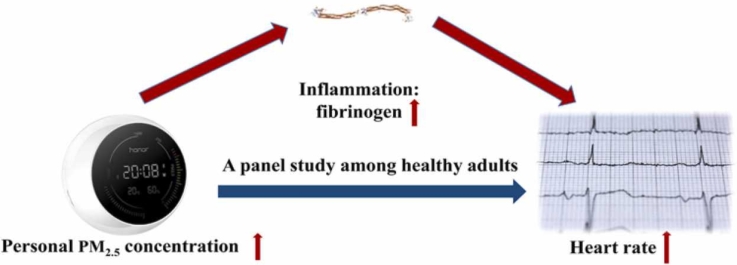

## 1 Introduction

Cardiovascular disease (CVD) has become a serious public health issue worldwide, causing 18.6 million deaths and 523 million prevalent cases worldwide in 2019 (Roth et al., 2020). In China, more than 29 million population suffer from cardiovascular diseases and the prevalence is increasing (Ma et al., 2020). Previous research has reported that exposure to particulate matter was significantly associated with CVD morbidity and mortality (Lelieveld et al., 2015, Patel et al., 2016, Rajagopalan et al., 2018). Fine particulate matter (PM_2.5_) could explain 11.7% of the total premature deaths from cardiovascular diseases in China in 2017(Yao et al., 2020). But how PM_2.5_ exposure can promote cardiovascular disease remained unconfirmed.

Heart rate (HR) and blood pressure (BP) are two independent indicators of cardiovascular function, and PM_2.5_ exposure can contribute to the incidence of CVDs (Brook et al., 2010, Woodward et al., 2014). In recent years, mounting evidence suggests positive associations of BP and HR with short-term PM_2.5_ exposure (Cakmak et al., 2014, Chang et al., 2015, Jacobs et al., 2012, Lin et al., 2017, Yang et al., 2019). However, other studies show null or even negative association in between (Baccarelli et al., 2011, Mirowsky et al., 2015, Ren et al., 2019, Xie et al., 2016, Zhang et al., 2020). In a recent meta-analysis involving 30 related studies, yang et al. concluded that an increment in PM_2.5_ (10 μg/m^3^) was associated with an elevation of 0.53 mmHg (95% CI: 0.26, 0.80) and 0.20 mmHg (95% CI: 0.02, 0.38) in systolic blood pressure and diastolic blood pressure, respectively (Yang et al., 2018). Evidence is inclusive concerning the relationship between the above-mentioned cardiovascular health indicators and PM_2.5_ exposure.

Previous studies showed that PM_2.5_ may affect cardiovascular functions via systemic inflammation, oxidative stress, autonomic nervous system imbalance and endothelial dysfunction (Bourdrel et al., 2017, Brook et al., 2010, Fiordelisi et al., 2017, Rajagopalan et al., 2018), but it has not been fully understood. High-sensitivity C-reactive protein (hs-CRP) is considered as a typical inflammation biomarker and can predict adverse cardiovascular outcomes (Stoner et al., 2013, Wang et al., 2021, Woods et al., 2000). Fibrinogen is a blood coagulation factor with inflammation properties, and it increases sensitively as the initiation of the inflammatory response (Hoppe, 2014). Epidemiological studies assessing the association of inflammation biomarkers and PM_2.5_ exposure were inconsistent. One meta-analysis including 22 related studies showed that fibrinogen increased by 3.51% per 10 μg/m^3^ increment in short-term PM_2.5_ exposure (Tang et al., 2020). Another systemic review on CRP indicated that PM exposure-induced CRP response varied among the different population and study designs (Li et al., 2012).

Therefore, we conduct a panel study among healthy young students in Wuhan to investigate associations of different PM_2.5_ levels with HR, BP, and hs-CRP and fibrinogen. Additionally, we further examined inflammation markers as mediators in the PM_2.5_-cardiovascular function association. The overarching goal is to elucidate how short-term exposure to PM_2.5_ affects cardiovascular function and clarify the potential mechanism.

## 2 Methods

### 2.1 Study design and subjects

From May 2019 to Jun 2019, we recruited 32 healthy adults from the School of Medicine, Wuhan University. Participants consisted of undergraduate and postgraduate students aged 20–29 years. Details of inclusive criteria and study design had been described previously (Liu et al., 2021). Briefly, we only included healthy people who had no cardiovascular disease or history of allergic disease. What’s more, the included participants should have stayed in Wuhan within the past three months. Each participant was exposed at two sites in Wuhan with different PM_2.5_ concentrations. The washout period between the two exposure sessions was two-week. The low-exposure region was the Moon Lake Park. It was located in the Hanyang District with a lake and a large number of trees surrounding it. While the high-exposure region was the Zhongyuan Square, which was a busy shopping area and close to heavy traffic in Qingshan District (Fig S1). Participants were asked to perform light-intensity activities like walking for 4 h (8:00 AM −12:00 PM) around the experiment sites. Participants commuted between Wuhan University and the experiment site by new-energy vehicles within 15 min. To avoid traffic-related air pollution exposure on the road, the car windows were kept closed. Basic information was collected using questionnaires during each visit. This work was approved by the Wuhan University Ethics Committee and each participant had provided written informed consent.

### 2.2 Exposure data

Each participant carried a HUAWEI individual PM_2.5_ monitor (Ai 100, Huawei Technologies Co., Ltd 2017, China) during each exposure session. The device was light-weighted to carry and could monitor personal PM_2.5_ concentrations, ambient temperature, and relative humidity in real-time. The detection range of this device is 0–250 μg/m^3^ and the detection accuracy is 1 μg/m^3^. Before the exposure sessions, we collocated the device with a DUSTTRAK™ DRX 8534 (TSI, USA) at the rooftop of a 4-story building for three days. The coefficient of determination (R^2^) was 0.94, showing that the device had good reliability and accuracy (Fig S2). Collected data can be checked and downloaded from an application on mobile phones by connecting the device with a phone using blue tooth. Hourly PM_2.5_ concentration, temperature, and relative humidity for each participant were downloaded by trained researchers after each exposure session.

### 2.3 Cardiovascular health indicators measurements

Before the measurement, we asked the participants to sit and rest for 5 min. During the walking period, the BP and HR of each participant were measured hourly using the OMRON electronic sphygmomanometer (China) on their right upper arm. BP was measured as millimeters of mercury (mmHg) and HR was measured as beats per minute (bpm). HR, systolic blood pressure (SBP), and diastolic blood pressure (DBP) could be read directly on the instrument, while mean arterial pressure (MAP) was obtained by DBP + 1/3 (SBP-DBP) and pulse pressure (PP) was the difference of SBP and DBP (Li et al., 2019).

### 2.4 Inflammation biomarkers measurements

After arriving at the School of Medicine, participants were gathered together immediately to draw blood in order by a phlebotomist at the lab. Plasma samples (10 mL) were collected in EDTAk2 anticoagulant tubes. After blood collection, EDTAk2 anticoagulant tubes were immediately mixed upside down for 5–6 times, so that the blood and anticoagulant were fully mixed before centrifugation. Serum samples (10 mL) were collected in non-anticoagulant tubes and set aside at room temperature (about 26 ℃) for half an hour and then blood samples (20 mL) were centrifuged at the speed of 3000 r/min for 10 min. Samples were separately aspirated into sterile dry lyophilization tubes and stored in a −80 ℃ refrigerator for subsequent analyses. Levels of serum hs-CRP were measured using the Au 5800 automatic biochemical analyzer (Beckman Coulter, US). The sensitivity of the assay was 0.06 mg/dL and the CV (coefficient of variation) was < 6%. The concentration of plasma fibrinogen was measured by automated blood clotting analyzer (Succeeder SF-8200, Beijing, China). The sensitivity of the assay was 0.8 g/L and the CV was < 5%.

### 2.5 Statistical analysis

Open-source R software (version 3.6.3, R Foundation for Statistical Computing) was used for all statistical analyses. The two-sided *p* < 0.05 was considered statistically significant. Continuous variables were described as means ± standard deviations (SD), and categorical variables were described as numbers and percentages (%). A paired *t*-test or Wilcoxon test was employed to compare variables of the two exposure sessions. Linear mixed-effect models were applied to fit the relationship of PM_2.5_ and health outcomes. The *lme4* package was used to fit the mixed-effect model (Bates et al., 2015). First, a crude model was established containing only PM_2.5_ concentrations, health outcomes, and participants’ ID. Then, in an adjusted model, we included sex, body mass index (BMI), age, temperature, and humidity as fixed effect covariates. We allowed random intercept for each participant and exposure session (1represents the low exposure session, 2 represents the high exposure session).

Considering the delayed health effect of PM_2.5_ exposure, we incorporated different lag intervals of PM_2.5_ exposure into the model. For BP and HR, PM_2.5_ concentration of lag 0 h, lag 1 h, lag 2 h, and lag 3 h were included in the model. Associations were expressed as estimated changes and 95% confidence interval (CI) of BP and HR by each 10 μg/m^3^ increment of PM_2.5_ concentrations. For inflammation biomarkers, PM_2.5_ concentration of 4 h average, lag 0–1 h, lag 2–3 h, and lag 0–3 h were included in the model. Levels of inflammation markers were normalized by natural logarithmic transformation. Effect estimates were described as percent changes with 95% CI in inflammation marker levels per 10 μg/m^3^ increase in PM_2.5_ concentrations.

Before mediation analysis, we explored the potential association between inflammation biomarkers, BP and HR. Inflammation biomarkers were included as exposure variables, while HR and BP as outcome variables in the adjusted model. We found that only HR was associated with fibrinogen. Therefore, we evaluated mediating effects of fibrinogen on the PM_2.5_ – HR association. We used the R package “*mediation”* to estimate the direct effects, average causal mediation effects (ACME), and total effect based on a widely-used method (Baron and Kenny, 1986). First, We developed a LMEM between fibrinogen and PM_2.5_ exposure. Then established another LME model linking heart rate with fibrinogen and PM_2.5_ exposure. The direct effect represented the direct effects of PM_2.5_ exposure on HR. The ACME represented the effects of fibrinogen(“mediator”) on the association of PM_2.5_ exposure (“treat”) and HR (“output”). The proportion mediated effect was how much fibrinogen could explain the association of PM_2.5_ exposure and HR. 100 times simulations of bootstrap analysis were performed to obtain the *p*-value of mediation proportion. Mediation models included the same covariates as the linear mixed-effect models. All variables were measured as 4 h average levels for consistency.

A sensitivity analysis was conducted to examine the robustness of results. According to the Chinese criterion of normal BMI, we excluded 5 participants whose BMI was over 23.9 kg/m^2^ and 3 participants whose BMI was below 18.5 kg/m^2^. The adjusted linear-mixed effect models were repeated by excluding participants mentioned above.

## 3 Results

### 3.1 Basic characteristics

We excluded one participant who withdrew after completing only one exposure session. Data from 62 valid samples were taken into the analyses. Among the 31 participants included, there were 11 males and 20 females; 22 were undergraduate and 9 postgraduate students. BMI ranged from 16 to 30 kg/m^2^ with an average of 21.8 (SD=3.4) kg/m^2^. The average age was 22.6 (SD=2.5) with a full range (20–29 years old). All the participants were non-smokers themselves, and most of them (93.8%) didn’t have alcohol intake during the experiment.

### 3.2 Exposure concentrations and biomarkers levels during the two different sessions

Table 1 described the air pollution, cardiovascular health indicators, and inflammation biomarkers of the two exposure sessions (low-exposure and high-exposure). PM_2.5_ concentration was significantly higher in the high-exposure session (68.33 μg/m^3^) than in the low-exposure session (10.28 μg/m^3^). The Wilcoxon test showed the differences in PM_2.5_ concentrations, temperature (29.31 ℃ vs. 31.22 ℃) and relative humidity (61.33% vs. 78.90%) of the two exposure sessions were statistically significant. There was no obvious difference among cardiovascular health indicators and inflammation biomarkers between the two exposure sessions.

**Table 1:** Average concentration of air pollution and biomarkers at two different exposure sessions.

Pollutants/Biomarkers	Low-exposure session	High-exposure session
(units)	Mean ± SD	Mean ± SD
PM_2.5_(μg/m^3^)*	10.28 ± 2.35	68.33 ± 8.82
Temperature(℃)*	29.31 ± 1.06	31.22 ± 1.52
Relative Humidity (%)*	61.33 ± 5.15	78.90 ± 2.01
*Cardiovascular health indicators*SBP(mmHg)	108.55 ± 13.01	106.96 ± 11.05
DBP(mmHg)	75.89 ± 10.81	74.24 ± 9.72
PP(mmHg)	32.67 ± 7.27	32.71 ± 6.66
MAP(mmHg)	86.78 ± 11.07	85.15 ± 9.68
HR(beats per min)	79.82 ± 9.32	84.79 ± 9.24
*Inflammation biomarkers*		
hs-CRP(mg/L)	1.10 ± 1.52	1.53 ± 2.69
Fibrinogen(g/L)	2.63 ± 0.38	2.63 ± 0.40

*Abbreviations*: SD, standard deviation; SBP, systolic blood pressure; DBP, diastolic blood pressure; PP, pulse pressure; MAP, mean artery pressure; HR, heart rate; hs-CRP, high-sensitivity C-reactive protein.

* Concentration difference of the variable was statistically significant.

### 3.3 The main findings

**Fig. 1:**
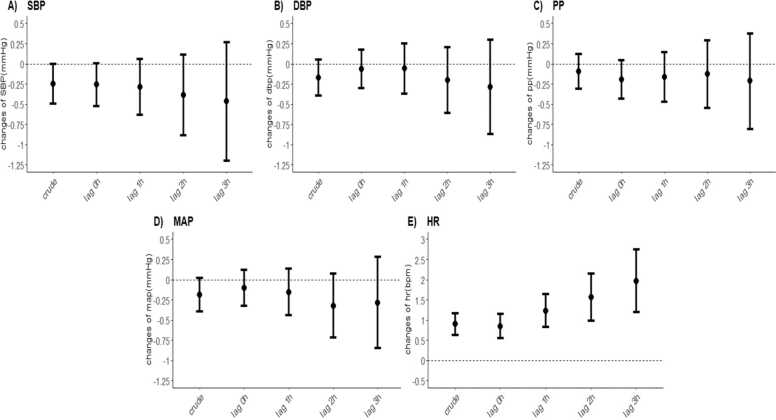
The association between four blood pressure components, heart rate, and short-term PM_2.5_ exposure. Changes (mean and 95% confidence intervals) in blood pressure and heart rate(E) associated with a 10 μg/m^3^ increase in PM_2.5_ exposure were estimated using different time lags. The blood pressure components included systolic blood pressure (SBP) (A), diastolic blood pressure (DBP) (B), pulse pressure (PP) (C) and mean artery pressure (MAP) (D). The adjusted models were adjusted for age, BMI, sex, temperature and relative humidity.

**Fig. 2:**
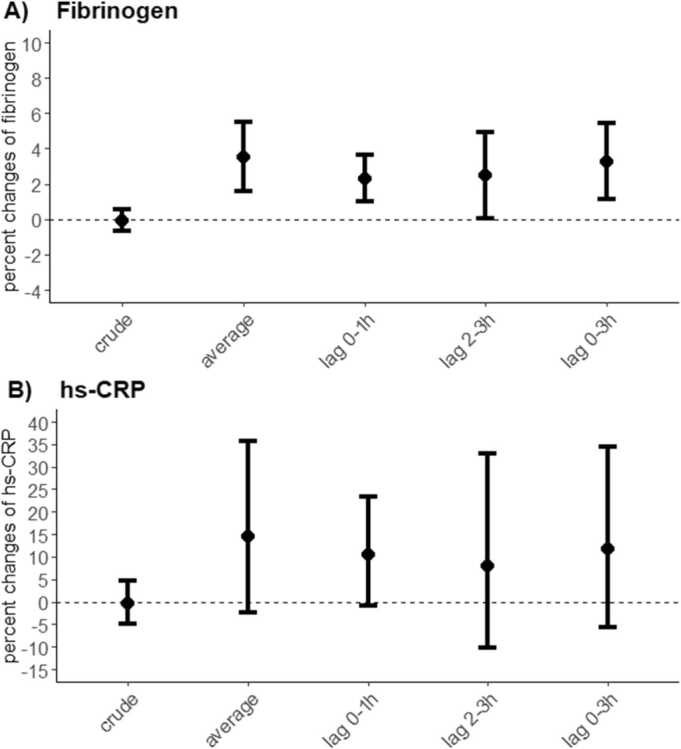
The association between related inflammation biomarkers and short-term PM_2.5_ exposure. Percent changes (mean and 95% confidence intervals) in inflammation biomarkers with 10 μg/m^3^ increases in PM_2.5_ were calculated at different time lag periods. Related inflammation biomarkers included Fibrinogen (A), and high-sensitivity C-reactive protein (hs-CRP) (B). The adjusted models were adjusted for age, BMI, sex, temperature and relative humidity.

Fig. 1 and Fig. 2 illustrated associations of air pollution with BP and HR and related inflammation biomarkers. in the crude model, changes in PM_2.5_ concentration were associated with decreased SBP and increased HR. After adjusted for the covariates, for each 10 μg/m^3^ increment in PM_2.5_ concentration, we observed an elevation of 0.84 bpm (95%CI: 0.54, 1.15) in HR and 3.52% (95% CI: 1.60%, 5.48%) in fibrinogen per 10 μg/m^3^ increase in PM_2.5_ concentration. In the lag effect model, the PM_2.5_ – HR association had an increasing trend with increasing lag intervals. The largest estimated change was at lag 3 h with an increase of 1.90 bpm (95% CI: 1.19, 2.75) in HR. For fibrinogen, we observed significant changes at multiple lag times. An increase in PM_2.5_ concentration(10 μg/m^3^) was associated with increased fibrinogen levels by 2.31% (95%CI: 1.03%, 3.64%) and 3.25% (95%CI: 1.13%, 5.47%) at lag 0–1 h and lag 0–3 h, respectively. Increased PM_2.5_ exposure elevated the concentrations of hs-CRP and lowered BP, though these changes were insignificant.

### 3.4 Mediation analysis

**Fig. 3:**
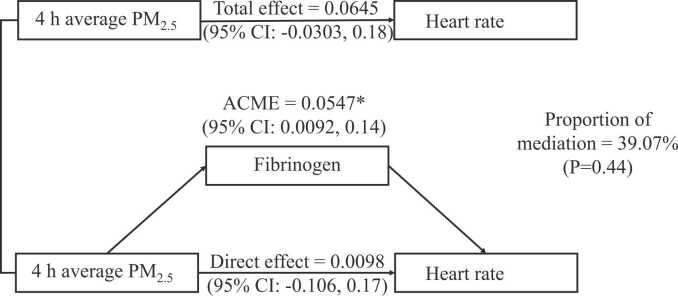
Mediation analysis of fibrinogen on heart rate after PM_2.5_ exposure. The mediation effect of fibrinogen between 4 h average PM_2.5_ exposure and heart rate were estimated. Average causal mediation effect (ACME), direct effect, total effect, and proportion of mediation were estimated with R package *mediation.* * the association was statistically significant.

Table 2 summarized the relationship of inflammation biomarkers with BP and HR. The results showed that every unit increase in fibrinogen was associated with an elevation in HR by 6.66 bpm (95%CI: 1.12, 12.38). However, we observed insignificant relationships between BP and inflammation biomarkers. The mediation analysis was described in Fig. 3. Only fibrinogen was included in the mediation analysis because it was associated with both PM_2.5_ exposure (the exposure) and HR (the outcome). The direct effect of PM_2.5_ exposure on HR was 0.01 (95%CI: −0.11, 0.17). The effect of fibrinogen on the association between PM_2.5_ exposure and HR was significant and the value of ACME was 0.05 (95%CI: 0.01, 0.14). Changes in fibrinogen could explain 39.07% (P = 0.44) of the increments in HR due to PM_2.5_ exposure.

**Table 2:** Associations of inflammation biomarkers with blood pressure and heart rate.

Variables		β (95%CI)	*p* value
fibrinogen			
	SBP	3.08 (−1.98, 8.73)	0.27
	DBP	-0.86 (−7.14, 5.08)	0.79
	PP	-2.80 (−6.59, 0.61)	0.14
	MAP	3.08 (−2.13, 8.64)	0.28
	HR	6.66 (1.12, 12.38)	0.03*
hs-CRP			
	SBP	-0.70(−1.56, 0.17)	0.13
	DBP	-0.35(−1.04, 0.41)	0.35
	PP	-0.38(−0.91, 0.07)	0.12
	MAP	-0.43(−1.15, 0.35)	0.27
	HR	0.78(−0.02, 1.67)	0.08

*Abbreviations*: SBP, systolic blood pressure; DBP, diastolic blood pressure; PP, pulse pressure; MAP, mean artery pressure; HR, heart rate; hs-CRP, high-sensitivity C-reactive protein; IL-6, Interleukin-6.

* The association was statistically significant.

### 3.5 The sensitivity analysis

As presented in Table 3, after excluding participants who were overweight (BMI > 23.9) or underweight (BMI < 18.5), the effect estimates of HR (0.85 bpm, 95%CI: 0.52, 1.18) and fibrinogen (4.03%, 95%CI: 1.78%, 6.21%) did not change substantially. Our results were robust by comparing the estimates of the main models and the sensitivity analyses.

**Table 3:** Changes in blood pressure, heart rate, and related inflammation biomarkers per 10 μg/m^3^ in PM_2.5_ exposure among participants whose BMI ranged from 18.5 to 23.9 kg/m^2^.

Variables	Changes (95%CI)	*p* value
fibrinogen	4.03% (1.78%, 6.21%)	0.00*
hs-CRP	12.61% (−6.05%, 35.52%)	0.24
SBP(mmHg)	-0.20 (−0.52, 0.10)	0.20
DBP(mmHg)	-0.09 (−0.36, 0.19)	0.54
MAP(mmHg)	-0.10 (−0.36, 0.15)	0.44
PP(mmHg)	-0.15 (−0.40, 0.11)	0.25
HR(bpm)	0.85 (0.52, 1.18)	0.00*

Changes were calculated for each 10 μg/m3 increment in fine particulate matter concentrations. 4 h average inflammation biomarkers, lag 0 h blood pressure, and heart rate were included in the sensitivity analysis.

* The association was statistically significant.

## 4 Discussion

In this panel study, we recruited 32 healthy adults and exposed them at two sites with different PM_2.5_ concentrations. One site was with PM_2.5_ concentrations of 68.33 ± 8.82 μg/m^3^ and the other site with 10.28 ± 2.35 μg/m^3^. We found that exposure to PM_2.5_, even for short periods(e.g., 4 h), was associated with elevated HR and activation of inflammation response. However, our finding was not enough to suggest that PM_2.5_ can affect HR via the inflammation pathway and no association was found between PM_2.5_ exposure with BP and hs-CRP.

This study showed the significant association between short-term PM_2.5_ exposure and elevated HR, which was consistent with previous research (Adar et al., 2007, Rich et al., 2012, Xie et al., 2016). A study showed that an IQR (interquartile range, 4.5 μg/m^3^) increment in the daily average PM_2.5_ concentration was associated with a 1.0% (95%CI: 0.9%, 1.2%) increase in HR among the elderly population(Adar et al., 2007). Lim et al. found that for each IQR increase in PM_2.5_ (13.45 μg/m^3^), HR increased 1.9 bpm (95%CI: 0.8, 3.0) among 466 elderly people living in Seoul, Korea (Lim et al., 2017). One study in Taipei asked healthy commuters to walk for 1 h and it was reported that HR increased by 3.4% (95%CI: 0.1, 3.5) for an IQR increase in PM_2.5_ (24 μg/m^3^) (Chuang et al., 2020). However, the null association was also reported by other studies between short-term PM_2.5_ exposure and HR (Cole-Hunter et al., 2018, Sun et al., 2013). The inconsistency might be due to different geographic regions, study populations, exposure metrics, PM_2.5_ sources, and constituents (Atkinson et al., 2014, Cai et al., 2016). Our findings showed the delayed effects of PM_2.5_ exposure on HR, which was reported by a previous study as well (Tsai et al., 2021). While we did observe the most pronounced effect at lag 3 h, the delayed period in this study was limited in light of the short exposure time.

Fibrinogen is a blood coagulation marker with pro-inflammation properties. It was shown that fibrinogen levels would increase in response to typical inflammation (Davalos and Akassoglou, 2012). This current study found short-term exposure to PM_2.5_ was positively associated with fibrinogen, which was in line with results reported by previous studies. A cross-sectional study based on the ESCAPE showed that fibrinogen increased 2.8% (95%CI: 0.5, 5.3) per 5 μg/m^3^ increment of PM_2.5_ concentrations (Lanki et al., 2015). Lee et al. reported that for each 10.4 μg/m^3^ increase in PM_2.5_ exposure, fibrinogen increased 0.44% (95%CI: 0.15%, 0.73%) among nonsmoking subjects (Lee et al., 2018). A meta-analysis showed that a 10 μg/m^3^ increment in short-term PM_2.5_ exposure was correlated with an increase of 0.54% (95% CI: 0.21, 0.86) in fibrinogen (Tang et al., 2020). Interestingly, percentage changes of fibrinogen level were modestly higher in our study (3.52%) than the ones listed above. A possible reason was that dose-response relationships between PM_2.5_ and health effects varied by region. We did find significant associations between fibrinogen and several lag intervals of PM_2.5_ exposure, but the largest change was observed for the current hour. Some studies found associations between PM_2.5_ exposure and fibrinogen with longer lag intervals. A quasi-experiment study during Beijing Olympics found 1.9% higher fibrinogen levels were associated with an IQR increase (6.5 μg/m^3^) of PM_2.5_ exposure at lag 3 days. (Rich et al., 2012). Tang et al. reported that a 10 μg/m^3^ increment in PM_2.5_ exposure 0.26% elevated fibrinogen levels by 0.26% (95%CI: 0.02%, 0.51%) at lag 1 day (Tang et al., 2020).

This study evaluated the role of fibrinogen in PM_2.5_ - HR association, though the mediated effect of fibrinogen was insignificance. Several studies explored the relationship between fibrinogen and HR and revealed that HR was significantly linked with fibrinogen levels (Jensen et al., 2012, Whelton et al., 2014). One of the Framingham heart studies showed that fibrinogen concentrations were 13% higher among women with fast heart rates ( 83.5 ± 7.7 bpm) as compared to those with low ones (53.7 ± 3.5 bpm) (Tofler et al., 2017). Another study found insignificant associations that an IQR (1.2 g/L) increase in fibrinogen was associated with 3.9% (95%CI: −3.7, 12.0) elevated HR (Luttmann-Gibson et al., 2010). However, few studies considered their association in the context of PM_2.5_ exposure. Theoretically, fibrinogen may be involved in two potential pathways that PM_2.5_ exposure affects HR. PM_2.5_ may contribute to coagulation abnormalities and accelerate thrombus formation by acting on coagulation factors (Bonzini et al., 2010, Brook et al., 2009). In addition, PM_2.5_ can activate systemic inflammation to exacerbate the imbalance of the cardio autonomic system (Croft et al., 2017). Overall, more epidemiological evidence about how circulating biomarkers function in the PM_2.5_-HR association is warranted to draw a confirmed conclusion.

There were some limitations in this study. First, we didn’t include covariates such as physical activity and dietary pattern of participants into our models, which may lead to residual confounding (El-Sayed et al., 2004, Zhong et al., 2017). However, since all the participants had their meal in the same school canteen, the dietary pattern would not vary so much between the two exposure sessions. Second, individual levels of gaseous pollutants (i.e. ozone and sulfur dioxide) were not monitored, which might adversely impact human health. For example, Hoffmann et al. reported that BP was inversely related to ozone but positively related to PM_2.5_ (Hoffmann et al., 2012).

## 5 Conclusions

Short-term exposure to PM_2.5_ was associated with elevated heart rate and increased fibrinogen levels. These relationships did not change substantially in participants with normal weight. Our finding was not enough to suggest that PM_2.5_ exposure can induce adverse cardiovascular effects by the pathway of inflammation. This study suggested that exposure to high PM_2.5_ concentrations even for 4 h could cause adverse effects on inflammation response and cardiovascular function. Regulations on PM_2.5_ concentrations should be considered by the government, especially in heavy traffic areas. Further studies in a larger population and different areas are warranted to confirm the acute effect of PM_2.5_ exposure on cardiovascular function and to elucidate the underlying mechanism.

## CRediT authorship contribution statement

**Zhaoyuan Li:** Data curation, Methodology, Formal analysis, Writing – original draft, Visualization. **Yisi Liu:** Validation, Writing – review & editing. **Tianjun Lu:** Validation, Writing – review & editing. **Shouxin Peng:** Methodology, Investigation, Data curation, Software. **Feifei Liu:** Investigation, Data curation, Software. **Jinhui Sun:** Investigation, Data curation, Software. **Hao Xiang:** Conceptualization, Methodology, Writing – review & editing, Funding acquisition.

## Declaration of Competing Interest

The authors declare that they have no known competing financial interests or personal relationships that could have appeared to influence the work reported in this paper.
