# Supplementary material for: Acute effect of fine particulate matter on blood pressure, heart rate and related inflammation biomarkers: A panel study in healthy adults
Source: Ecotoxicol Environ Saf. 2021 Dec 25;228:113024. doi: 10.1016/j.ecoenv.2021.113024 (PMC8655618; doi:10.1016/j.ecoenv.2021.113024)
Supplement: Supplementary material [file mmc1.docx]

**Supplemental Material**

1.
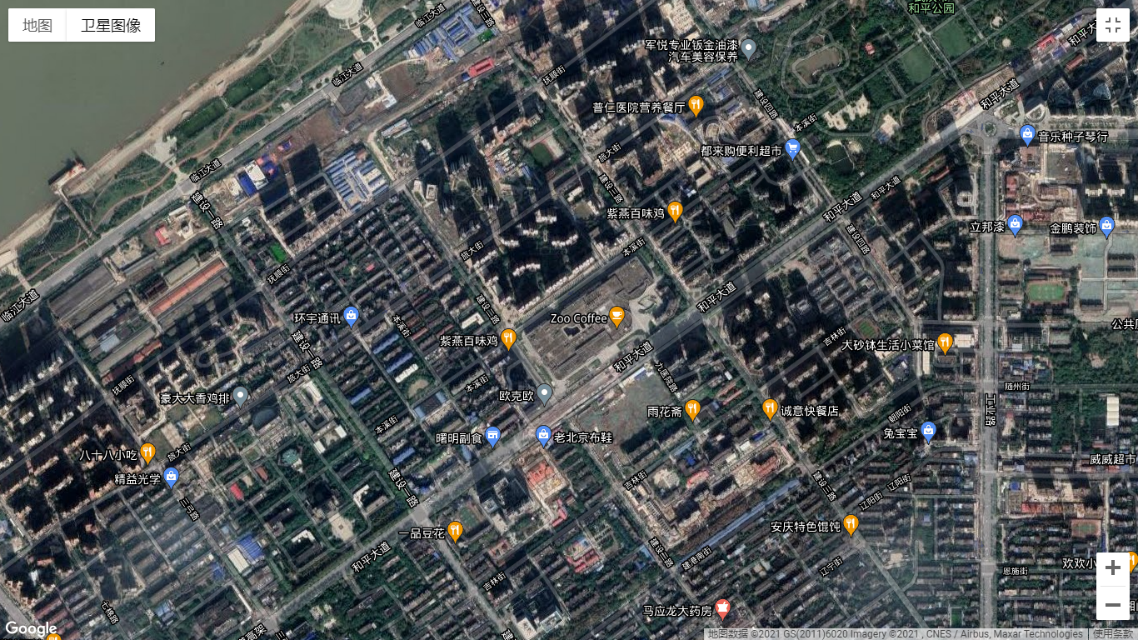
The Zhongyuan Square
2.
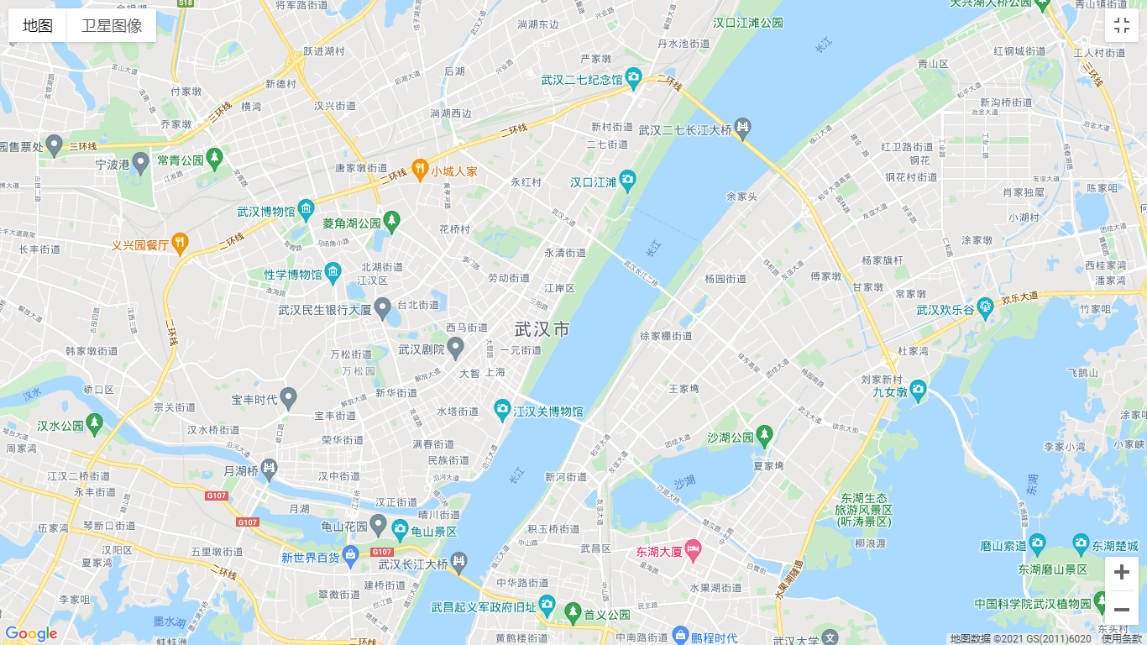

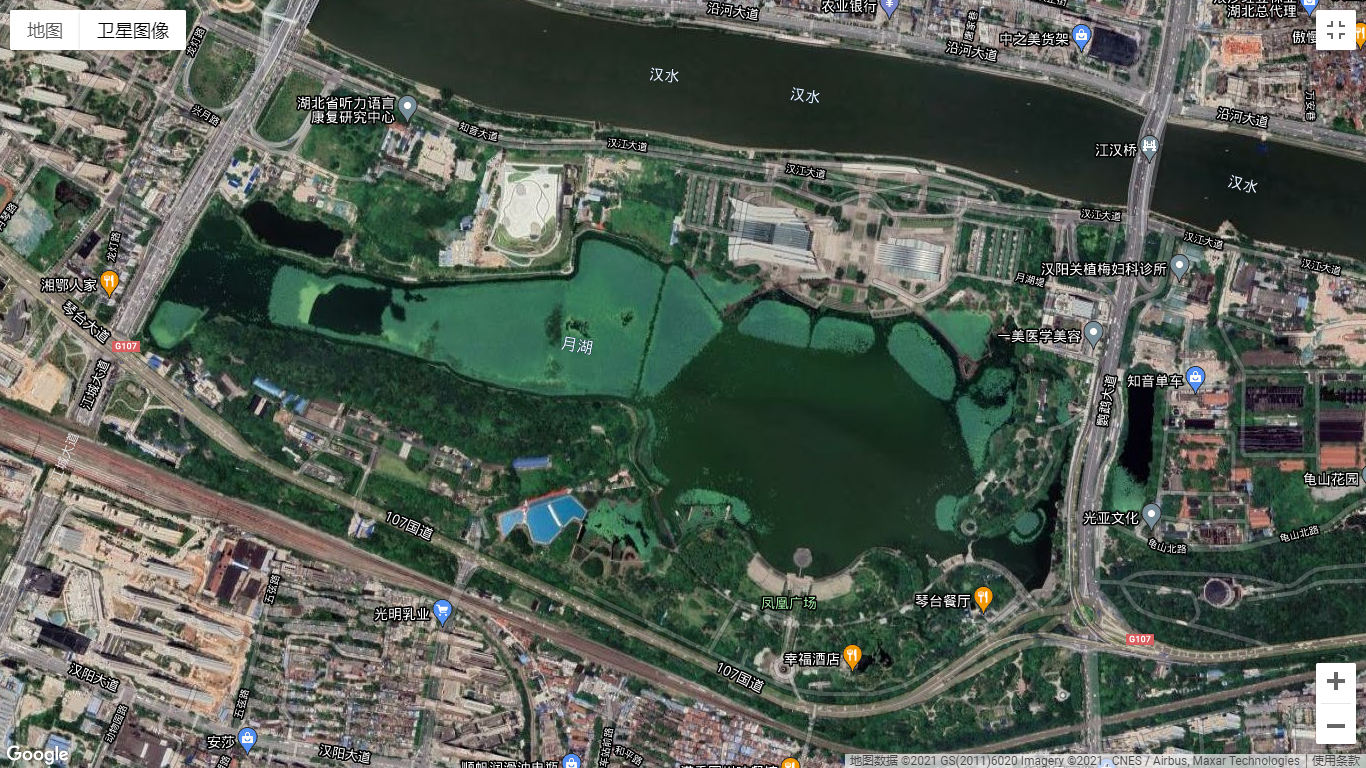
The Moon Lake Park

The Moon Lake Park

(30.55°N, 114.26°E)

The Zhongyuan Suqare

(30.62°N, 114.38°E)

Fig S1. The satellite images of two experiment sites (A. The Zhongyuan Square; B. The Moon Lake Park) in this panel study and their location in Wuhan city (C). Data was obtained from the online Google Maps (http://www.gditu.net/).

Fig S2. The association between PM_2.5_ concentrations from HUAWEI individual PM_2.5_ monitor and TSI 8534. Data was collected every hour from 18:00 April 15^th^ to 18:00 April 17^th^ in 2019. The 4-story building was the main teaching building located in the school of medicine, Wuhan University. A linear regression was used to assess the correlation between data from TSI 8534 and HUAWEI individual PM2.5 monitor.
